# The Relationship between Type D Personality and Depression Symptoms in Stroke Patients: The Chain Mediating Effect of Self‐Efficacy and Participation Preferences

**DOI:** 10.1002/brb3.71209

**Published:** 2026-01-28

**Authors:** Huipin Zhang, Suying Yu, Yun Ye

**Affiliations:** ^1^ Department of Neurology The First People's Hospital of Changzhou and the third Affiliated Hospital of Soochow University Changzhou China; ^2^ Department of Nursing The First People's Hospital of Changzhou and the third Affiliated Hospital of Soochow University Changzhou China

**Keywords:** chain mediation, depression symptoms, self‐efficacy, stroke, Type D personality

## Abstract

**Background:**

Stroke significantly impacts population health, and post‐stroke depressive symptoms are highly prevalent. Although depression symptoms are linked to Type D personality, self‐efficacy, and participation preferences in discharge planning, the mechanisms underlying these interactions remain unclear. This study aimed to examine the chain mediation effects of self‐efficacy and discharge planning participation preferences on the relationship between Type D personality and depression symptoms in stroke patients.

**Methods:**

This study used a convenience sampling method to recruit 318 stroke patients from the Department of Neurology at the First People's Hospital of Changzhou. Participants were assessed using the Type D personality scale (DS14), stroke self‐efficacy questionnaire (SSEQ), patient participation preferences assessment (PPPA), and self‐rating depression scale (SDS). Statistical analyses included descriptive analysis, Spearman's rank correlation analysis, and chain mediation analysis; these analyses were performed using SPSS 25.0 and PROCESS v3.5 (Model 6).

**Results:**

A total of 311 stroke patients were included in this study. Type D personality, self‐efficacy, participation preferences, and depression symptoms are significantly correlated with each other (all *P* < 0.001). Self‐efficacy and participation preferences acted as significant mediators between Type D personality and depression symptoms. The total indirect effect accounted for 46.51% of the total effect, and the chain pathway contributed 7.23% (chain indirect effect = 0.030, 95% CI 0.014–0.050).

**Conclusion:**

Type D personality indirectly influences depression symptoms through the chain mediation effects of self‐efficacy and participation preferences in stroke patients. This study demonstrates the intrinsic mechanisms by which Type D personality contributes to depression symptoms, providing insights for healthcare professionals to prevent and clinically intervene in stroke patients with depression.

## Graphical abstract

Type D personality is associated with depression symptoms in stroke patients. In 311 inpatients, a chain mediation model identified a significant chain mediating effect of self‐efficacy and discharge planning participation preferences on this association. The total indirect effect accounted for 46.51% of the total effect, and the chain pathway contributed 7.23% (chain indirect effect = 0.030, 95% CI 0.014‐0.050). Enhancing self‐efficacy and discharge engagement may help reduce depression symptoms.

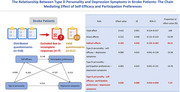

## 1 Introduction

Accelerated population aging and changes in lifestyle have increased exposure to risk factors for stroke, thereby elevating the disease burden. Stroke has become the world's second leading cause of death and third leading cause of disability (He et al. 2024), with its mortality impact projected to remain persistently high through 2050 (GBD 2021 Forecasting Collaborators 2024). Characterized by high incidence, recurrence, disability, and mortality rates along with substantial economic burden, stroke severely impacts global physical and mental health.

Post‐stroke depression symptoms are highly prevalent among stroke patients. Studies have reported that the prevalence of post‐stroke depression ranges from 34.3% to 42.3% (Liu et al. 2024; Zheng et al. 2025; Tinsae et al. 2025). Separately, one study reported a depression rate of 31.0% within five years after stroke (Hackett and Pickles 2014). Depression symptoms reduce quality of life (Gurková et al. 2023), double suicide risk compared to the general population (Hong et al. 2018), and increase all‐cause mortality by 1.59‐fold (Cai et al. 2019). Consequently, early screening and interventions are critical (Dai et al. 2024).

Numerous studies have investigated risk factors for depression symptoms, with growing attention on Type D personality (also termed “distressed personality”), characterized by negative affectivity and social inhibition. A prospective observational cohort study confirmed that Type D personality is a significant predictor of depression (Yin et al. 2023), establishing a causal relationship. Studies demonstrate that self‐efficacy is correlated with Type D personality and depression symptoms (Shao et al. 2017; Wu et al. 2015; Gao et al. 2022). This suggests interconnections among Type D personality, self‐efficacy, and depression symptoms.

Bandura's self‐efficacy theory posits that self‐efficacy determines behavioral motivation and outcomes through individuals’ subjective assessments of their capability to execute specific actions (Bandura 1977). Those with high self‐efficacy tend to undertake appropriately challenging tasks and confront difficulties proactively. Schwarzer's health action process approach theory similarly demonstrates that self‐efficacy promotes health behaviors by influencing behavioral intentions (Schwarzer 2014). Both theories suggest correlations between patients’ self‐efficacy and their preferences for engaging in specific actions. Given that stroke patients often experience residual dysphagia (Wen et al. 2025), language impairments, cognitive deficits, and hemiplegia (Gunaratne et al. 2023)—complicating home‐based care—discharge planning is critically important (Osborne et al. 2023). This process encompasses pre‐discharge assessments and post‐discharge care coordination to ensure continuity of medical services (Goncalves‐Bradley et al. 2022). Patient participation preferences in discharge planning reflect behavioral intentions (Huber and McClelland 2003), suggesting potential correlations between self‐efficacy and participation preferences.

Our synthesis shows: (1) Type D personality predicts depression symptoms; (2) self‐efficacy mediates the relationship between Type D personality and depression symptoms; and (3) self‐efficacy correlates with patient participation preferences. These findings suggest interactions among these four variables. Elucidating the pathogenic mechanisms connecting Type D personality to depression symptoms poses major challenges for developing interventions in stroke patients. Early identification and mitigation of depression symptoms are essential, yet few studies have empirically validated these hypotheses using theoretical models. We consequently hypothesize that self‐efficacy and participation preferences exert chain mediation effects between Type D personality and depression symptoms in stroke patients.

## 2 Methods

### 2.1 Study Design

This single‐center, observational cross‐sectional study employed convenience sampling to survey stroke inpatients in the Department of Neurology at the First People's Hospital of Changzhou between October 2024 and February 2025. The hospital is a Class A tertiary comprehensive institution that integrates healthcare, teaching, and research, with 2980 licensed beds and an annual discharge of 154,900 patients.

### 2.2 Participants

Using Monte Carlo power analysis, anticipated standardized path coefficients were *a_1_* = –0.3, *d_21_* = 0.3, and *b_2_* = –0.3, yielding an expected serial indirect effect of *a_1_* × *d_21_* × *b_2_* = (−0.30) × 0.30 × (−0.30) = 0.027, two‐tailed *α* = 0.05, target power = 0.8; the Monte Carlo simulation indicated a minimum effective sample size of approximately *N* = 135 under the web‐app's default assumptions (Schoemann et al. 2017, [Link].).

The inclusion criteria for participants were: (1) clinically diagnosed stroke; (2) age ≥ 18 years; (3) clear consciousness with normal communication ability; and (4) willingness to participate in data collection. Exclusion criteria comprised: (1) major organ failure (e.g., heart, lungs, liver, kidneys, etc.); (2) pre‐existing psychiatric disorders; and (3) ICU admission.

### 2.3 Ethics Statement

This study strictly adhered to the principles of the Declaration of Helsinki and was approved by the ethics committee of the First People's Hospital of Changzhou, No: 2022 (Ke) 143. Before data collection, researchers fully informed participants about the study objectives and procedures. All participants provided written informed consent voluntarily. Researchers rigorously followed ethical principles, immediately terminating surveys if participants withdrew consent.

### 2.4 Measurement

#### 2.4.1 Covariates

Covariates were determined through literature review and research team discussions, including: (1) gender (male/female); (2) age (18–59 years/ ≥ 60 years); (3) monthly income (<1000 CNY/1000–3000 CNY/ > 3000 CNY); and (4) self‐rated health (good/fair/poor).

#### 2.4.2 SSEQ

This scale was originally developed by Jones et al. (Jones et al. 2008) in 2008 and subsequently translated and culturally adapted by Li et al. (Li et al. 2015) in 2015, and it should be assessed as soon as the patient is stable. SSEQ comprises two dimensions: activities of daily living efficacy (6 items) and self‐management efficacy (5 items), totaling 11 items. Responses are recorded on a 0–10 scale, yielding a total score range of 0–110. Higher scores indicate greater confidence and capability in rehabilitation exercises among stroke patients. Cronbach's α coefficients ranged between 0.942 and 0.974 for both total and subscales (Li et al. 2015).

#### 2.4.3 PPPA

This scale was originally developed by McClelland et al. (McClelland and Kelly 1980) in 1980 and culturally adapted by Wu et al. (Wu et al. 2024) in 2024, it is recommended to administer it prior to patient discharge. PPPA comprises three dimensions: communication and participation (8 items), willingness and preference (5 items), and desire for information (4 items), totaling 17 items. Each item employs a 5‐point Likert scale ranging from 1 (not at all) to 5 (very much), with total scores spanning 17–85. Higher scores indicate stronger patient participation preferences in discharge planning. The total scale demonstrated a Cronbach's α of 0.958 and test‐retest reliability of 0.929 (Wu et al. 2024).

#### 2.4.4 DS14

This scale was developed by Denollet et al. and revised in 2005 (Denollet 2005), then introduced to China by Yu et al. in 2006 (Yu and Zhang 2006). It comprises two dimensions: social inhibition and negative affectivity, each containing seven items (14 items total). Using a 5‐point Likert scale (0 = not at all, 4 = very true), Type D personality is diagnosed when both dimensions score ≥ 10. Higher scores indicate stronger Type D personality tendencies. Psychometric validation in the Taiwan region of China demonstrated Cronbach's α coefficients of 0.86 for negative affectivity and 0.79 for social inhibition (Weng et al. 2013). Test‐retest reliability for both dimensions was 0.76 and 0.74, respectively (Yu et al. 2010).

#### 2.4.5 SDS

This scale was originally developed by Zung (Zung 1965) in 1965 and introduced to China by Wang et al. (Wang and Chi 1984) in 1984, and it is used to assess the patient's current status or status during the past week. The 20‐item scale employs a 4‐point Likert format (1 = not at all/rarely, 4 = most/all of the time). The raw score is obtained by summing all item scores. The standard score is calculated by multiplying the raw score by 1.25 and retaining the integer portion. A standard score ≥ 53 indicates depressive symptoms, with higher scores reflecting greater symptom severity. The scale demonstrates a Cronbach's α coefficient of 0.81 (Tanaka‐Matsumi and Kameoka 1986).

### 2.5 Procedure

Two researchers trained in standardized protocols conducted all surveys. They used a uniform script to explain the study objectives and allow participants to self‐determine involvement. The instructions clearly stated that the survey was anonymous, that all data would be used solely for academic purposes, and would be kept strictly confidential. To psychologically separate the measurement tasks for different constructs, this study provided independent instructions for each core variable to guide participants in switching their mental context. Furthermore, in the questionnaire presentation, we intentionally used different Likert scale ranges and inserted brief transition sentences between modules (e.g., “Next, please consider another aspect”) to create visual and psychological breaks, thereby reducing participants’ response inertia. Literate participants without reading difficulties completed the questionnaires independently; others orally narrated the questionnaire items, one by one, in a neutral tone. We strictly preserved the reverse‐scored items included in the original scale development. During data cleaning, these items were reverse scored. Because these items are semantically opposite or different from other items in the same scale, they effectively interrupt participants’ unconscious response sets, compelling them to read and understand the meaning of each item rather than mechanically selecting answers in the same direction, thereby enhancing data validity and diversity. Researchers immediately reviewed completed questionnaires for missing responses and rectified omissions. All documents were stored in sealed plastic containers to ensure data confidentiality and integrity.

### 2.6 Data Analysis

Data entry was performed using Microsoft Excel 2016. Statistical analyses were conducted using SPSS version 25.0 (IBM Corp., Armonk, NY, USA). Qualitative data are presented as frequencies and percentages. Normally distributed quantitative data are expressed as mean ± standard deviation, while non‐normally distributed data are described using median and the interquartile range (IQR). We assessed common method bias using Harman's single‐factor test. Kolmogorov–Smirnov tests revealed non‐normal distributions for SDS, SSEQ, and DS14 scores, whereas PPPA scores followed a normal distribution. The demographic characteristics of stroke patients were compared using the Mann‐Whitney U test, independent samples *t*‐test, Kruskal‐Wallis test, and one‐way ANOVA. Pairwise correlations were analyzed using Spearman's rank correlation. Statistical significance was set at *p* < 0.05 (two‐tailed). Despite the presence of non‐normally distributed data, in large samples (*N* > 30), parameter estimates remain robust based on the Central Limit Theorem, and chain mediation analysis can be performed (Field 2024, Ibrahim et al. 2025). Chain mediation analyses (Model 6, PROCESS v3.5) specified SDS scores as the dependent variable (Y), DS14 scores as the independent variable (X), SSEQ scores as the first mediator (M1), and PPPA scores as the second mediator (M2). Covariates included gender, age, monthly income, and self‐rated health. We employed the bootstrap method (5000 iterations) to estimate the indirect effects and their 95% confidence intervals (CIs), which do not rely on the assumption of normal data distribution (Shrout and Bolger 2002). Effects were considered statistically significant if the 95% CIs excluded zero.

## 3 Results

### 3.1 Common Method Bias Test

Common method bias was assessed using Harman's single‐factor test (Wang et al. 2024). Exploratory factor analysis incorporating all 62 items from four scales (SSEQ, PPPA, DS14, and SDS) revealed 14 factors with eigenvalues >1. The first factor accounted for 24.56% of variance, below the 40% threshold criterion, suggesting the absence of significant common method bias.

### 3.2 Descriptive Analysis

A total of 318 questionnaires were distributed in this study and 7 were excluded due to incomplete responses, yielding 311 valid questionnaires (valid response rate 97.80%). Among the 311 stroke patients in this study, 192 (61.74%) were male and 119 (38.26%) were female. The mean age was 67.32 ± 9.68 years. Monthly income distribution showed: <1000 CNY (*n* = 14, 4.50%), 1000–3000 CNY (*n* = 43, 13.83%), and > 3000 CNY (*n* = 254, 81.67%). Self‐rated health status was reported as good (*n* = 85, 27.33%), fair (*n* = 189, 60.77%), or poor (*n* = 37, 11.90%). Type D personality was identified in 65 patients (20.90%), while 95 (30.55%) screened positive for depression symptoms. The scale scores were as follows: DS14 scores median 16.00 (IQR 9.00–22.00), SDS scores median 48.00 (IQR 40.00–54.00), SSEQ scores median 87.00 (IQR 65.00–102.00), and PPPA scores mean 59.49 ± 9.46. See Table 1 for details.

**TABLE 1:** Descriptive statistics of study variables (*N* = 311).

Scale	Central tendency	Min‐max
DS14	Median 16.00 (IQR 9.00–22.00)	0–50
SSEQ	Median 87.00 (IQR 65.00–102.00)	2–110
PPPA	Mean 59.49 ± 9.46	34–81
SDS	Median 48.00 (IQR 40.00–54.00)	28–79

Abbreviatons: DS14 = Type D personality scale, SSEQ = stroke self‐efficacy questionnaire, PPPA = patient participation preferences assessment, SDS = self‐rating depression scale. Values are presented as median (interquartile range, IQR) or mean ± standard deviation, as appropriate.

Significant differences in DS14 scores were observed by gender (*p* < 0.05). Significant differences in SSEQ scores were observed by age and self‐rated health status (*p* < 0.05). No significant differences in PPPA scores were found across demographic variables (*p* > 0.05). Significant differences in SDS scores were observed by age and self‐rated health status (*p* < 0.05). See Table 2 for details.

**TABLE 2:** Differences in variable scores among different demographic characteristics of stroke patients (*N* = 311).

Variable	*n* (%)	Type D personality	Self‐efficacy	Participation preferences	Depression symptoms
Gender	—	—	—	—	—
Male	192 (61.74)	18.00 (10.00, 23.00)	86.00 (64.00, 102.00)	58.71 ± 9.63	48.50 (40.50, 55.00)
Female	119 (38.26)	13.00 (9.00, 20.00)	90 (70.00, 103.00)	60.74 ± 9.08	45.00 (40.00, 53.00)
*Z*/*t* value	—	−2.745	−1.085	−1.847	−1.699
*p* value	—	0.006	0.278	0.066	0.089
Age (yr)	—	—	—	—	—
18–59	59 (18.97)	14.00 (7.00, 21.00)	97.00 (82.00, 106.00)	61.00 (55.00, 67.00)	44.00 (39.00, 50.00)
≥ 60	252 (81.03)	16.00 (10.00, 22.00)	85.00 (64.00, 100.00)	59.00 (53.00, 66.00)	48.00 (42.00, 55.00)
*Z* value	—	−1.159	−3.108	−1.339	−2.744
*p* value	—	0.247	0.002	0.181	0.006
Monthly income (CNY)	—	—	—	—	—
<1000	14 (4.50)	17.00 (10.00, 27.00)	77.00 (70.00, 96.00)	56.29 ± 9.31	52.00 (44.00, 56.00)
1000—3000	43 (13.83)	18.00 (12.00, 27.00)	90 (61.00, 103.00)	58.33 ± 9.24	48.00 (40.00, 58.00)
>3000	254 (81.67)	15.00 (8.00, 21.00)	87.50 (67.00, 102.00)	59.86 ± 9.50	48.00 (40.00, 53.00)
*H/F* value	—	5.481	0.988	1.323	2.284
*p* value	—	0.065	0.610	0.268	0.319
Self‐rated health	—	—	—	—	—
Good	85 (27.33)	16.00 (10.00, 24.00)	94.00 (80.00, 104.00)	59.00 (54.00, 68.00)	44.00 (39.00, 51.00)
Fair	189 (60.77)	15.00 (9.00, 21.00)	86.00 (64.00, 102.00)	61.00 (53.00, 66.00)	49.00 (43.00, 54.00)
Poor	37 (11.90)	17.00 (12.00, 26.00)	77.00 (51.00, 100.00)	63.00 (50.00, 67.00)	50.00 (41.00, 61.00)
*H* value	—	4.823	8.893	0.101	13.721
*p* value	—	0.090	0.012	0.951	0.001

Type D personality was assessed using the Type D personality scale; self‐efficacy was assessed via the stroke self‐efficacy questionnaire; participation preferences were assessed with the patient participation preferences assessment; and depressive symptoms were measured using the self‐rating depression.*H* values are from the Kruskal–Wallis test; *t* values are from the independent samples *t*‐test; *Z* values are from the Mann–Whitney U test; *F* values are from one‐way ANOVA. A *p* value < 0.05 was considered statistically significant.

### 3.3 Correlation Analysis of Self‐Efficacy, Participation Preferences, Type D Personality, and Depression Symptoms

There were significant positive correlations among Type D personality, self‐efficacy, participation preferences, and depression symptoms in stroke patients (*p* < 0.001 for all pairs). See Table 3.

**TABLE 3:** Correlation analysis of self‐efficacy, participation preferences, Type D personality, and depression symptoms (*r*, *N* = 311).

Variable	Type D personality	Self‐efficacy	Participation preferences	Depression symptoms
Type D personality	1.000	—	—	—
Self‐efficacy	−0.268	1.000	—	—
Participation preferences	−0.388	0.463	1.000	—
Depression symptoms	0.413	−0.579	−0.509	1.000

Type D personality was assessed using the Type D personality scale; self‐efficacy was assessed via the stroke self‐efficacy questionnaire; participation preferences were assessed with the patient participation preferences assessment; and depressive symptoms were measured using the self‐rating depression scale. All Spearman correlation analyses showed *p* < 0.001.

### 3.4 Analysis of Chain Mediation Effects of Self‐Efficacy and Participation Preferences Between Type D Personality and Depression Symptoms

**FIGURE 1:**
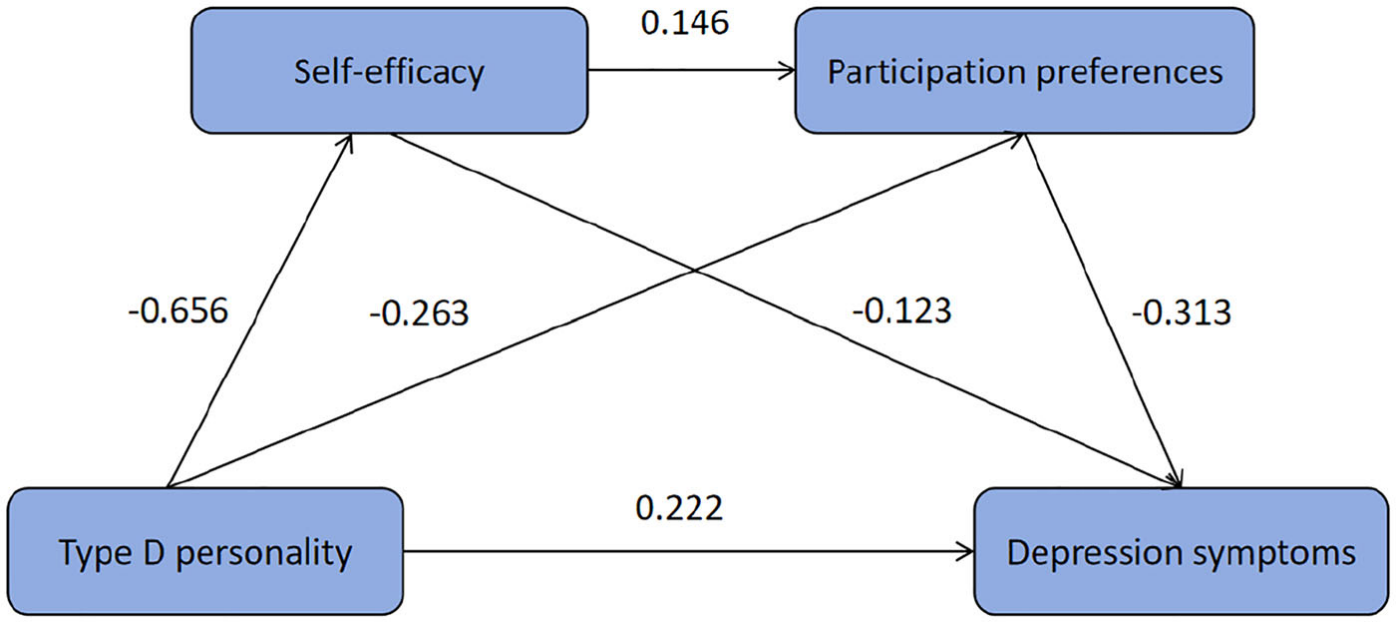
Schematic diagram of chain mediation effects of self‐efficacy and participation preferences on the association between Type D personality and depression symptoms in stroke patients. All coefficients are unstandardized coefficients; the solid line represents the statistically significant path (*p* < 0.05).

Results demonstrated significant mediation effects: the indirect effect through self‐efficacy alone was 0.080, through participation preferences alone was 0.082, and the indirect effect through both mediators was 0.030 (95% CI: 0.014–0.050). The total effect was 0.415 (95% CI: 0.314–0.517), and the direct effect was 0.222 (95% CI: 0.131–0.314). See Tables 4, 5 and Figure 1 for details.

**TABLE 4:** Analysis of chain mediation effects of self‐efficacy and participation preferences between Type D personality and depression symptoms (*N* = 311).

Outcome variable	Prediction variable	Regression coefficient	*SE*	*t*	*p*	*R^2^*	*F*	*p*
Self‐efficacy	Type D personality	−0.656	0.144	−4.570	<0.001	0.118	8.167	<0.001
Participation preferences	Type D personality	−0.263	0.050	−5.275	<0.001	0.290	20.647	<0.001
—	Self‐efficacy	0.146	0.019	7.600	<0.001	—	—	—
Depression symptoms	Type D personality	0.222	0.046	4.786	<0.001	0.481	40.116	<0.001
—	Self‐efficacy	−0.123	0.019	−6.555	<0.001	—	—	—
—	Participation preferences	−0.313	0.051	−6.130	<0.001	—	—	—

Type D personality was assessed using the Type D personality scale; self‐efficacy was assessed via the stroke self‐efficacy questionnaire; participation preferences were assessed with the patient participation preferences assessment; and depressive symptoms were measured using the self‐rating depression scale. Statistical significance is indicated by the *t* value and *p* value. The overall model fit is indicated by the coefficient of determination (R^2^), and the overall significance of the model is assessed by the *F*‐statistic (*F*) test. A *p* value < 0.05 was considered statistically significant.

**TABLE 5:** Examination of chain mediation effects of self‐efficacy and participation preferences on the association between Type D personality and depression symptoms in stroke patients (*N* = 311).

Path	Effect value	*SE*	95% CI	Proportion of effect value (%)
Total effect	0.415	0.051	0.314—0.517	100
Direct effects	0.222	0.046	0.131—0.314	53.49
Indirect effects	0.193	0.033	0.132—0.262	46.51
Type D personality—self‐efficacy—depression symptoms	0.080	0.022	0.041—0.129	19.28
Type D personality—participation preferences—depression symptoms	0.082	0.023	0.041—0.131	19.76
Type D personality—self‐efficacy—participation preferences—depression symptoms	0.030	0.009	0.014—0.050	7.23

Type D personality was assessed using the Type D personality scale; self‐efficacy was assessed via the stroke self‐efficacy questionnaire; participation preferences were assessed with the patient participation preferences assessment; and depressive symptoms were measured using the self‐rating depression scale. Abbreviatons: *SE* = standard error; CI = confidence interval.

## 4 Discussion

To our knowledge, this is the first study to validate the chain mediation effects of self‐efficacy and participation preferences between Type D personality and depression symptoms in stroke patients. The findings demonstrate that Type D personality influences depression symptoms by reducing self‐efficacy and thereby attenuating engagement in discharge planning. This not only advances our understanding of the relationship between Type D personality and depression symptoms in stroke populations but also offers innovative frameworks for preventing and clinically managing depression symptoms.

### 4.1 Type D Personality and Depression Symptoms

This study confirms that Type D personality in stroke patients predicts depression symptoms, consistent with prior research (Yin et al. 2023). People with Type D personality exhibit chronically elevated negative affectivity, blunted heart rate responses, and lower blood pressure, indicating inadequate acute stress responses. Their psychological and interpersonal vulnerabilities promote problem avoidance, increasing susceptibility to depression and suicide risk (Kupper et al. 2013, 2023, Denollet et al. 2021). Metabolomic analyses reveal overlapping metabolite biomarkers for negative affectivity in Type D personality and depression symptoms, suggesting shared pathophysiological mechanisms (Li‐Gao et al. 2025). Additionally, inflammatory responses may mediate the Type D personality‐depression relationship. Chronic self‐imposed stress in Type D people triggers inflammatory cascades, upregulating pro‐inflammatory cytokines (van Dooren et al. 2016). Activated immune cells release mediators (e.g., IL‐1β, IL‐6, and IL‐18), while sustained inflammation impairs transcription of neurotrophic factors and reduces neuroplasticity in the prefrontal cortex and hippocampus (Yamanishi et al. 2022, Medeiros et al. 2020). Early evaluation of Type D personality in stroke patients is therefore critical, enabling targeted interventions to reduce the occurrence of depression symptoms.

### 4.2 Mediation Effects of Self‐Efficacy and Participation Preferences

This study confirms the mediating role of self‐efficacy in the relationship between Type D personality and depression symptoms, indicating that Type D personality reduces self‐efficacy in stroke patients, thereby promoting depression symptoms. People with Type D personality exhibit pessimistic cognitive biases, fixating on negative aspects of situations while inhibiting emotional expression in social contexts. This makes the feeling of failure and inadequacy persist (Shao et al. 2017). Their personality traits diminish social support and self‐efficacy, leading to reduced rehabilitation confidence, diminished life engagement, and impaired sense of self‐worth due to physical limitations and stroke‐related complications — all contributing to depression symptoms (Chau et al. 2021). Kandola et al. (Kandola et al. 2019) demonstrated that physical activity mitigates depression, but sustainable behavioral change requires enhanced self‐efficacy (Bandura 1977), informing targeted interventions. These results suggest that enhancing self‐efficacy may reduce the impact of Type D personality on depressive symptoms.

We further validate the mediating effect of patient participation preferences between Type D personality and depression symptoms. Type D personality significantly reduces proactive engagement in discharge planning, elevating risk of depression symptoms. This may stem from patients' pessimistic outcome expectations, where they perceive discharge planning as futile and disengage due to preemptive failure assumptions. Grounded in Bandura's social cognitive theory, reframing patients' perceptions through observational learning of successful rehabilitation cases may counteract pessimistic expectations, leveraging outcome expectations as motivational drivers (Bandura 2014).

### 4.3 The Chain Mediation Model

This study validates the chain mediation effects of self‐efficacy and participation preferences between Type D personality and depression symptoms in stroke patients. It reveals a progressive pathway in which Type D personality triggers diminished self‐efficacy, then weakened participation in discharge planning, ultimately elevating depression symptoms. This mechanism may stem from Type D people's negative affectivity, fostering adverse outcome expectations, while social inhibition limits exposure to peer success stories, synergistically eroding rehabilitation confidence. Patients with low self‐efficacy lack perceived control and confidence in discharge planning, reinforcing failure anticipation and avoidance of personalized goal‐setting. Passively accepting standardized discharge protocols often leads to implementation barriers due to patient‐provider misalignment, increasing risks of complications and readmission—further validating negative self‐perceptions and activating depression (Lin et al. 2024). Thus, concurrently targeting self‐efficacy and participation preferences disrupts this pathological cascade, establishing an effective clinical framework to reduce depression symptoms incidence.

### 4.4 Clinical Implications

This study's chain mediation model not only clarifies the psychological mechanisms by which Type D personality contributes to depressive symptoms, but also offers a practical framework for early identification of high‐risk patients in clinical settings. For newly admitted stroke patients, Type D personality should be assessed with the DS14 as soon as the patient's condition stabilizes. Particular attention should be given to patients with low stroke rehabilitation self‐efficacy and those who demonstrate limited engagement in discharge planning. Targeted interventions for them may help interrupt the subsequent maladaptive pathway and ultimately reduce the risk of depressive symptoms in this population.

The findings of this study suggest that interventions aimed at improving rehabilitation self‐efficacy and engagement in discharge planning may serve as effective strategies to reduce the impact of Type D personality on depressive symptoms. First, research indicates that digital self‐management has a certain effect on improving self‐efficacy in stroke patients, suggesting that healthcare providers can guide patients in adopting digital self‐management interventions to enhance self‐efficacy and further mitigate the development of depression. It is recommended to integrate digital tools with routine care to address challenges related to patients' limited digital literacy (Guo et al. 2025). Second, older age, lower education levels, and more negative attitudes predict lower participation preference, indicating that healthcare professionals should pay closer attention to patients with these characteristics. Approaches such as empowerment education and guiding patients to participate in online self‐help groups may help improve participation preference, thereby reducing depressive symptoms (Büdenbender et al. 2023). Third, a randomized controlled trial shows that virtual reality (VR) intervention can effectively alleviate negative emotions in patients with Type D personality, suggesting that healthcare professionals may use VR‐based interventions to distract patients from negative thoughts and stimuli, thereby mitigating the adverse emotions associated with Type D personality and subsequently relieving depressive symptoms (Wu et al. 2025). It should be noted, however, that the implementation of VR interventions faces limitations, such as the additional cost of VR equipment, low acceptance among older patients, and the need for well‐designed content. Future efforts should promote the adoption of VR through financial, personnel, and technical support.

## 5 Limitations

This study has several limitations. First, as this was a cross‐sectional study, the data cannot establish causal relationships. Moreover, because the SDS and DS14 relies on retrospective self‐report of past experiences or states, responses may be subject to recall and social desirability biases, which may compromise measurement accuracy. Future longitudinal studies and interventional studies should validate sequence‐mediated pathways and validate causality. Second, sampling from a single hospital limits generalizability. Multicenter studies with larger cohorts are needed to enhance external validity. Finally, although we adjusted for key covariates, residual confounding may persist. Further exploration of critical unmeasured confounders is essential to verify result robustness.

## 6 Conclusions

This study validates the association between Type D personality and depression symptoms, along with the chain mediation effects of self‐efficacy and participation preferences in this pathway. Our findings provide critical insights for depression symptoms prevention and management, highlighting that interventions should prioritize enhancing self‐efficacy and discharge planning engagement in stroke patients. Future research should explore additional mediators to comprehensively elucidate the pathogenic mechanisms linking Type D personality to depression symptoms development, ultimately improving patient outcomes.

## Nomenclature

CI: Confidence Interval
DS14: Type D Personality Scale
IQR: Interquartile Range
PPPA: Patient Participation Preferences Assessment
SSEQ: Stroke Self‐Efficacy Questionnaire
SDS: Self‐Rating Depression Scale
VR: Virtual Reality

## Author Contributions

Huipin Zhang and Yun Ye designed the study; Huipin Zhang wrote the manuscript; Suying Yu and Yun Ye revised the manuscript. All authors read and approved the final manuscript.

## Funding

This work was supported by Changzhou Commission of Health under the Top Talent of Changzhou “The 14th Five‐Year Plan” High‐Level Health Talents Training Project, No. 2022260.

## Ethics Statement

This study strictly adhered to the principles of the Declaration of Helsinki and received ethical approval from the Institutional Review Board of the First People's Hospital of Changzhou, No. 2022 (Ke) 143. Participation was voluntary, and written informed consent was obtained from all participants. All the data were anonymized during analysis.

## Conflicts of Interest

The authors declare no conflicts of interest.

## Data Availability

The datasets used or analyzed during the current study are available from the corresponding authors on reasonable request.
